# A Dream Problem

**Published:** 1915-11

**Authors:** 


**A Dream Problem.** A correspondent of Pract. Med., Delhi, Ind., Sept., asks for help in the following case. A man has repeatedly a dream of addressing an audience, the subject of his address being that the whole thing is a dream, including the audience who, in the dream, deride him for not recognizing that the dream is a real occurrence and not a dream. He wishes help to convince his imaginary audience that it is imaginary. This is a logical extension of Freudism. It is high time that the medical profession should cultivate the lost function of hard-headedness.

